# Evaluating common drivers for color, iron and organic carbon in Swedish watercourses

**DOI:** 10.1007/s13280-014-0560-5

**Published:** 2014-11-15

**Authors:** Johan Temnerud, Julia K. Hytteborn, Martyn N. Futter, Stephan J. Köhler

**Affiliations:** 1 Department of Aquatic Sciences and Assessment, Swedish University of Agricultural Sciences, P.O. Box 7050, 750 07 Uppsala, Sweden; 2 Department of Earth Sciences, Uppsala University, P.O. Box 256, 75105 Uppsala, Sweden

**Keywords:** Iron, Organic carbon, Organic matter, pH, Water color, Drinking water production

## Abstract

The recent browning (increase in color) of surface waters across much of the northern hemisphere has important implications for light climate, ecosystem functioning, and drinking water treatability. Using log-linear regressions and long-term (6–21 years) data from 112 Swedish watercourses, we identified temporal and spatial patterns in browning-related parameters [iron, absorbance, and total organic carbon (TOC)]. Flow variability and lakes in the catchment were major influences on all parameters. Co-variation between seasonal, discharge-related, and trend effects on iron, TOC, and absorbance were dependent on pH, landscape position, catchment size, latitude, and dominant land cover. Large agriculture-dominated catchments had significantly larger trends in iron, TOC, and water color than small forest catchments. Our results suggest that while similarities exist, no single mechanism can explain the observed browning but show that multiple mechanisms related to land cover, climate, and acidification history are responsible for the ongoing browning of surface waters.

**Electronic supplementary material:**

The online version of this article (doi:10.1007/s13280-014-0560-5) contains supplementary material, which is available to authorized users.

## Introduction

Browning of the waters has been reported for many lakes and rivers in the northern hemisphere (Forsberg and Petersen 1990; Roulet and Moore 2006; Monteith et al. 2007). This increase in water color changes lake light climate (Karlsson et al. 2009) and thermal regime (Snucins and Gunn 2000) and potentially affects food web structure (Wallace et al. 1997). Roulet and Moore (2006) reviewed the science associated with browning and linked it solely to increase in surface water dissolved organic matter concentrations. Iron (Fe) was not mentioned as a contributing factor although the knowledge that both iron and organic matter contribute to water color is old (Eriksson 1929; Åberg and Rodhe 1942; also see Kritzberg and Ekström 2012). The traditional paradigm that increasing organic matter, expressed as total organic carbon (TOC, see Box 1 for explanations of acronyms used in the article) concentrations, would lead to browner waters may not always hold, since in-lake processes tend to selectively remove the highly colored fraction of TOC that strongly binds Fe (Köhler et al. 2013).

**Box 1:** Explanation of acronyms used in the article

Acronym	Definition
1 − *β*	Statistical power analysis, the probability of correctly accepting the null hypothesis
AbsF	Absorbance at 420 nm (5 cm cuvette) of a filtered sample (0.45 µm), this is equivalent to true water color
AbsF_mgPtL_	AbsF converted to color expressed as platinum concentrations: Color = 500 AbsF
AbsUnF	Absorbance at 420 nm (5 cm cuvette) in an unfiltered water sample. This parameter includes the light absorbing parameters of dissolved, colloidal and particulate material
AbsUnF_mgPtL_	AbsUnF converted to platinum concentrations: Color = AbsUnF 500
*a*_0_	a_0_ is the constant in the log-linear model (Eq. 1)
*a*_1_	a_1_ is the discharge coefficient (Eq. 1)
*A*	*A* is the amplitude of seasonality (Eq. 1)
*c*	*c* is an offset controlling the timing of peaks in seasonality (Eq. 1)
*a*_4_	*a*_4_ is the trend coefficient (Eq. 1)
DOC	Dissolved organic carbon filtered at 0.45 µm
FA	Common factor analysis
Fluxmaster	A system for log-linear regression modeling (Schwarz et al. 2006)
HYPE	Daily discharge data based on Hydrological Predictions for the Environment model (Lindström et al. 2010)
Limes Norrlandicus	A physical geographic border/zone in Sweden with significant differences north and south of the border in air temperature, precipitation (duration of snow cover), vegetation (e.g., boreal and taiga vegetation north of the border) and soil type, etc. (Sernander 1901)
NSE	Nash–Sutcliffe efficiency index (Eq. 2 in Nash and Sutcliffe 1970)
PCA	Principal component analysis
PLS	Partial least squares regression
PTHBV	Daily air temperature and precipitation, spatially interpolated data on a 4x4 km grid (Johansson 2002)
*Q* ^2^	The goodness-of-fit parameter used in PLS, which is the average (n = 7, default value in SIMCA) explained variance of a randomly selected fraction (1/n of the data) of data not used to fit the model
SEPA	Swedish Environmental Protection Agency
SIMCA	SIMCA for Windows v13.0 (Umetrics), software for PLS
TOC	Total Organic Carbon
VIP	Variable importance on the projection, is the relative importance of each X variable ranked using VIP scores, derived as the sum of square of the PLS weights across all components
wq	Water quality (i.e., absorbance, Fe and TOC)

While color, Fe, and TOC co-vary, previous studies have suggested that they are not synchronous (Kritzberg and Ekström 2012; Weyhenmeyer et al. 2012). Since TOC and Fe, and thus water color, are influenced by pH, sulfate and alkalinity can also be important controlling factors (Neal et al. 2008; Sjöstedt et al. 2010; Knorr 2013). Much of the recent observed increase in TOC in many surface waters in the northern hemisphere has been ascribed to a recovery from acidification and subsequent increase in pH (Vuorenmaa et al. 2006; Monteith et al. 2007). Furthermore, the formation of particulate Fe in surface waters is strongly pH dependent (Köhler et al. 2013; Neubauer et al. 2013). Thus, it is likely that the increase in pH associated with recovery from acidification has had an impact on distribution of dissolved and particulate Fe which, in turn, will influence water color.

Understanding color, Fe, and TOC, seasonal dynamics and their relationship to discharge may provide important insights into current climatic controls on water quality and hence the possibility of browner water in the future. This knowledge is needed for future proofing drinking water treatment plants and for assessing possible changes in aquatic ecology.

Here, we present an analysis of long-term (6–21 years) time series of color, Fe, and TOC from 112 watercourses sampled in the Swedish long-term surface water monitoring program. We used routine monitoring data on four water quality parameters: total organic carbon concentrations (TOC), total iron (Fe), filtered (AbsF = water color), and unfiltered (AbsUnF) absorbance at 420 nm to identify and better understand spatial patterns in the co-variation of water color-related parameters in Swedish watercourses spanning a range of gradients related to catchment size, latitude, elevation, and land use/land cover.

We used nonlinear regression modeling to identify discharge, seasonal, and long-term trend components in the individual time series for each watercourse. Relationships between regression coefficients for each parameter and catchment characteristics were evaluated using weighted regressions and multivariate methods.

Our predictions are that

1. The values (concentration) in AbsF, AbsUnF, Fe, and TOC display positive co-variation.
2. This co-variation (1) is explained by similar mobilization mechanisms that are related to discharge and seasonality.
3. Longer water retention time is consistent with selective removal of AbsF, AbsUnF, Fe, and TOC, which will dampen and weaken the co-variation.
4. There are differences in co-variation between (i) North and South Sweden and (ii) large and small catchments.

## Materials and methods

### Study area and data

**Fig. 1:**
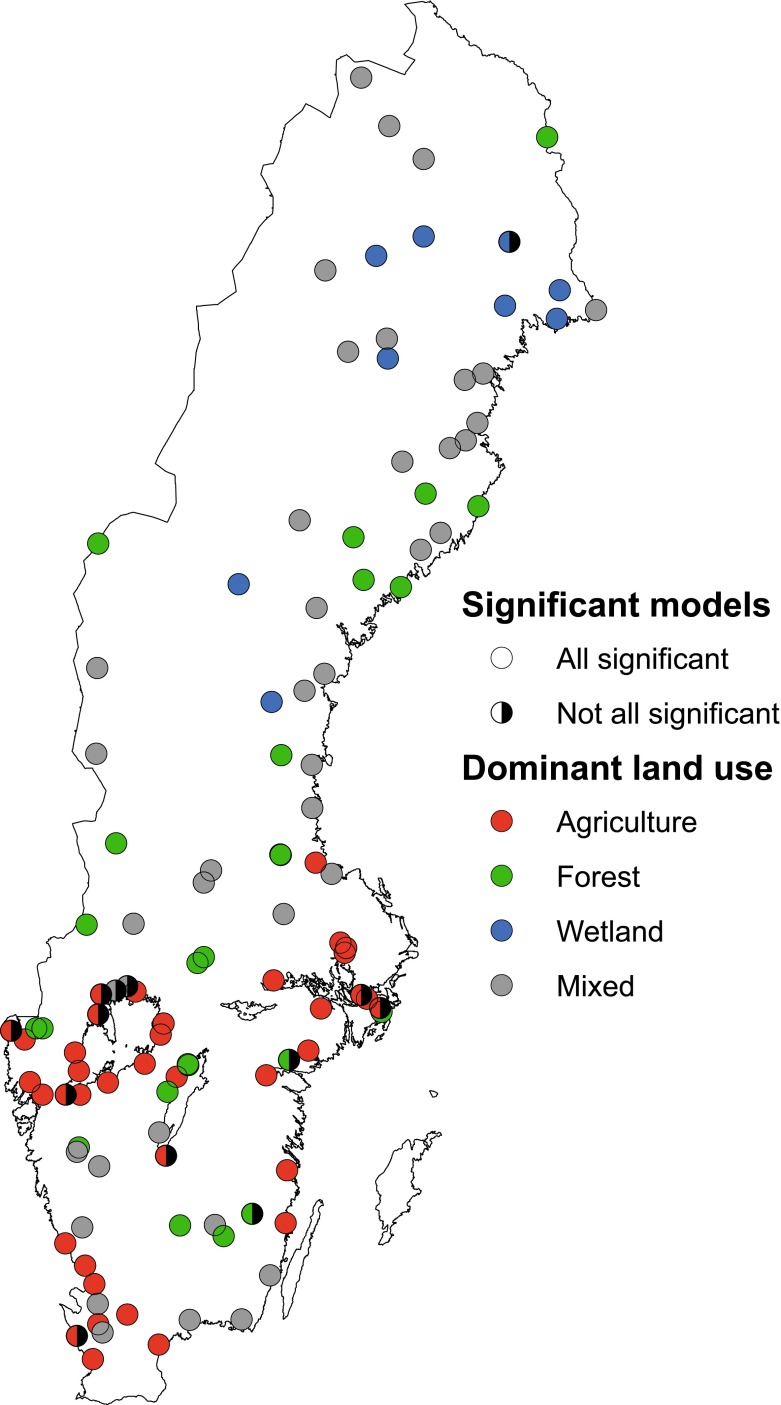
Map of sampling locations. The *colors* on the *dots* in the graph represent land use (*red* for agriculture, *green* for forest, *blue* for wetland, and *black* for mixed land use)

TOC, Fe, AbsF, and AbsUnF from 112 watercourses were included in this study. Monthly data series of at least 6 years duration for all four water quality parameters were used. The median time series length was 15 years between 1990 and 2010. Within each watercourse, the four water quality parameters had the same start and end date. The range in average AbsF was (0.015 to 0.57 420 nm 5 cm^−1^), AbsUnF (0.019 to 0.83 420 nm 5 cm^−1^), Fe (0.04 to 3.3 mg L^−1^), and TOC (1.4 to 24 mg L^−1^). Average values for alkalinity varied between −0.08 and 3.8 meq L^−1^, pH from 4.5 to 7.9, and sulfate from 0.02 to 1.3 meq L^−1^. The watercourses were from all across Sweden and their locations ranged from latitude 55°–68°N, which are over 1400 km (Fig. 1). There is an important north–south environmental gradient in Sweden known as the Limes Norrlandicus. This is a physical geographic border/zone in Sweden with significant differences north and south of the border in air temperature, precipitation (duration of snow cover), vegetation (e.g., boreal and taiga vegetation north of the border) and soil type (Sernander 1901).

The water chemistry data came from the national database at the Swedish University of Agricultural Sciences (SLU) collected within the national environmental monitoring program (Fölster et al. 2014). All samples were analyzed at one laboratory at the Department of Aquatic Sciences and Assessment, using Swedish Board for Accreditation and Conformity Assessment accredited methods (SLU 2011).

TOC was measured as CO_2_ after combustion using a Shimadzu TOC-VPCH analyzer, after acidification and sparging to remove inorganic carbon. Dissolved organic carbon (DOC) is the concentration of organic carbon in a filtered (common cutoff is 0.45 µm filter) water sample. It has previously been shown that DOC and TOC differ on average by less than 5 % (Ivarsson and Jansson 1994; Köhler 1999), so TOC is essentially identical to DOC in the large majority of the Swedish surface waters (see also Gadmar et al. 2002). Iron was analyzed using ICP-AES on unfiltered samples, while absorbance was measured at a single wavelength of 420 nm on filtered (0.45 µm) and unfiltered water samples. So as to compare the different water quality parameters in the log-linear models (the constant term, see the next paragraph), AbsF and AbsUnF were recalculated to concentration platinum equivalents using the equation Color = 500·Absorbance (SEPA 2007) and are hereafter referred to as AbsF_mgPtL_ and AbsUnF_mgPtL_. In the absence of turbidity which is a widely used measure for quantifying amounts of particulate matter, we used AbsUnF as a surrogate for turbidity in this study. Turbidity is known to co-vary with suspended matter including silt, clay, or other mineral particles such as Fe or Al (Spellman and Drinan 2012). Turbidity was introduced into the national monitoring program in 2010 and is not part of the present data series. Time series plots from all 112 watercourses were manually inspected, and a total of 12 Fe, three TOC, one AbsUnF, and no AbsF exceptionally high outliers values were removed (no watercourses were removed), from the total dataset (*n* = 22 780).

The daily discharge data used in the regression model came from the hydrological predictions for the environment (HYPE) model (Lindström et al. 2010) developed at the Swedish Meteorological and Hydrological Institute. Catchment characteristic data were used to evaluate potential causes of variability in the water quality parameters. Catchment area and mean elevation were taken from the Swedish national 50 m grid database. Land use data were obtained from the CORINE database (Bossard et al. 2000), soil type data from the Geological Survey of Sweden, soil map and forest data came from the kNN database (Reese et al. 2003) which contains data about the average age of the forest, biomass volume, and stand species composition. Daily air temperature and precipitation came from the PTHBV-database (Johansson 2002). The PTHBV-database contains spatially interpolated data on a 4 × 4 km grid, from mostly Swedish meteorological stations. Catchment specific daily mean temperature and precipitation values were calculated based on the grid points in each catchment.

### Log-linear regression of AbsF, AbsUnF, Fe, and TOC

Daily AbsF_mgPtL_, AbsUnF_mgPtL_, Fe, and TOC concentrations were estimated with the Fluxmaster system for log-linear regression modeling (Cohn et al. 1992; Schwarz et al. 2006). In the results presented here, one regression model was estimated for each station and water quality parameter. So as to systematically compare the patterns of water quality parameters in different watercourses, the same model structure was used for each parameter in all 112 watercourses. The model included discharge, seasonality (sine term with the period of one year and an arbitrary offset), and a long-term trend, see Eq. 1:

1 $$ \ln \left({\text{wq}} \right) = {a}_{0} + {a}_{1} \ln\left({\text{Discharge}} \right) + {A}\sin \left({2\pi {\text{dtime}} + {c}} \right) + {a}_{4} {\text{dtime}} $$

wq is one of AbsF_mgPtL_, AbsUnF_mgPtL_, TOC, and Fe concentration. *a*_0_, *a*_1_, *A*, *c*, and *a*_4_ are model coefficients, ln(Discharge) is the natural logarithm of daily discharge, and dtime is decimal years. Furthermore, a_0_ is the constant, a_1_ is the discharge coefficient, *A* is the amplitude of seasonality, *c* is an offset controlling the timing of peaks in seasonality, and *a*_4_ is the trend coefficient (Hytteborn et al., unpublished results). The model coefficients for the four different water quality parameters were compared to identify similarities and differences between watercourses.

Figure S1 (Electronic Supplementary Material) illustrates the four separate model terms that together make up the modeled values for AbsF_mgPtL_ at one site. The figure is in log-space, and the model and all terms are significant (*P* < 0.05) for this watercourse (River Nissan, Halmstad). All four terms have an impact on AbsF_mgPtL_ in this watercourse, values of AbsF_mgPtL_ display seasonality with a peak in September and a clear influence from the discharge. There was also a trend in both the observed and modeled data.

### Statistical analyses

In order to evaluate co-variation in the water quality parameters, the data were divided into classes (Table 1). The choice of variables followed most of the well-known major landscape and chemical drivers of water chemistry such as sulfate, acidity (pH), buffer capacity (alkalinity), theoretical water retention time (Müller et al. 2013), and land use. Classification and regression tree analysis was used on median values of AbsF to generate land use classes based on percentages of agricultural, forest, lakes, and wetland land cover (De’ath and Fabricius 2000).

**Table 1:** Different classes used in the study and color codes used in figures. The two first columns are connected; otherwise, there is no correlation between the different columns

Land use	Color code	Catchment size (km^2^)	pH	SO_4_ (meq L^−1^), median	Alkalinity (meq L^−1^), median
Agriculture >5 %	Red	>0.2, <20	<6.0	<0.1	≤0
Forest >80 %	Green	>21, <624	≥6.0	>0.1, <0.5	>0, <1
Wetland >20 %	Blue	>725, <3442		>0.5	>1
Mixed	Black	>3,590			

In order to evaluate the model performance, the *r* ^2^, which is the proportion of the water quality parameter explained by the *x*-variables, and the Nash–Sutcliffe efficiency index (NSE) was computed (Eq. 2 in Nash and Sutcliffe 1970). The NSE ranges between −∞ and 1. A NSE-value of 0 means that the model is equally good as the mean of the observed data and a NSE-value of 1 represent a perfect fit between the modeled and observed data.

Multiple linear regressions were used to explain long-term median values of AbsF_mgPtL_ using Fe and TOC from each site. Weighted linear regression was performed on amplitudes (*A*, Eq. 1), the model coefficient derived from the four water quality parameters. The data were divided into pH, sulfate, and alkalinity classes (Table 1) to see if they differed in their behavior. The weights in the regression were the sum of *r* ^2^ from the two compared Fluxmaster models (Fe and TOC)—amplitudes from a model with high *r* ^2^ had more influence in the regression and significant Fluxmaster models.

Differences between class results were assessed using post hoc statistical power analysis (see Electronic Supplementary Material). The statistical power (1 − *β*) gives the probability that the hypothesis is correctly rejected; a value above 0.80 indicates that the null hypothesis of no difference between classes was correctly rejected.

Both common factor analysis (FA) and partial least squares regression (PLS) were used to assess relationships between measured water quality parameters and model coefficients (see Electronic Supplementary Material).

## Results

Out of the 112 watercourses, 99 had significant log-linear models for all four water quality parameters. Significant models were obtained for AbsF_mgPtL_ and AbsUnF_mgPtL_ in 110 watercourses (median *r* ^2^ = 0.51 and 0.54, median NSE = 0.48 and 0.46, respectively); TOC in 106 watercourses (*r* ^2^ = 0.46, NSE = 0.43) and 104 watercourses had significant Fe models (*r* ^2^ = 0.41, NSE = 0.35). The Fluxmaster log-linear models worked best for AbsF_mgPtL_ and AbsUnF_mgPtL_, while Fe models were generally poorer. In the 25 % of the largest catchments (area >3590 km^2^), the weakest Fe models were obtained with both *r* ^2^ and NSE significantly (1 – *β* > 0.80) lower than for AbsF_mgPtL_ and AbsUnF_mgPtL_. According to NSE, log-linear models for AbsF_mgPtL_ and AbsUnF_mgPtL_ worked significantly (1 − *β* > 0.80) better than that for Fe for all catchment sizes (Table 2). Strong correlations were observed between AbsF_mgPtL_ and TOC for both *r* ^2^ and NSE, at all area classes, and both north and south of Limes Norrlandicus.

**Table 2:** Mean values in log-linear model coefficients. The 99 watercourses where all four water quality parameters have significant models are shown. Measured is observation with absorbance data multiplied with 500 to get data in mg Pt L^−1^. NSE is Nash–Sutcliffe efficiency index. See the “Materials and methods” section for more details

	*N*	Measured (mg L^−1^)	Constant	Discharge	Trend	Amplitude	Peak day (Julian)	*r* ^2^	NSE
AbsF	99	90	2.8	0.25	0.010	0.25	198	0.52	0.47
AbsUnF	99	125	3.1	0.23	0.010	0.25	196	0.53	0.47
Fe	99	0.73	4.9	0.15	0.0085	0.30	177	0.41	0.34
TOC	99	10	1.3	0.15	0.011	0.18	217	0.47	0.44

### Measured water quality parameters

Based on all 112 watercourses, there was a significant (1 − *β* > 0.95) difference in measured mean AbsUnF and TOC concentrations for north and south of Limes Norrlandicus (AbsUnF 94 and 151 mg Pt mg L^−1^; TOC of 7.4 and 12 mg L^−1^, respectively). For AbsF and Fe, statistical powers to detect differences were 1 − *β* = 0.73 and 0.23, respectively. When comparing the different area (catchment size) classes, AbsF_mgPtL_, AbsUnF_mgPtL_, and TOC had significant differences in the mean between the smallest (0.2–20 km^2^; Table 1) and largest (>3590 km^2^) catchments as well as for the second largest catchments (725–3442 km^2^) vs the largest catchments. For both AbsF_mgPtL_ and AbsUnF_mgPtL_, a significant difference between second smallest catchments (21–624 km^2^) vs the largest catchments was noted. For Fe, the highest mean concentration was observed for the second smallest catchments, as opposed to smallest catchments as for the other three water quality parameters. We also observed a decreasing mean concentration with increasing catchment area for Fe (the second smallest catchments vs the largest had 1 − β of 0.74). Spearman ranking (also indicated by Pearson and partial correlations) gave stronger correlations between AbsF_mgPtL_ versus TOC (0.95) and Fe (0.88) for north of Limes Norrlandicus than to the south (0.80 and 0.77, respectively). For the smaller catchments (<624 km^2^), Spearman rank correlations were higher between AbsF_mgPtL_ and TOC than AbsF_mgPtL_ and Fe. For the two larger catchments size classes, (>725 km^2^) AbsF_mgPtL_ and Fe had stronger correlations.

### The constant coefficient

Most results for the constant coefficients are similar to measured water quality parameters. The mean constant coefficient of the 99 watercourses with significant log-linear models differed significantly (1 − β > 0.95) between all four water quality parameters, except between AbsF_mgPtL_ (2.8) and AbsUnF_mgPtL_ (3.1) (Table 2). There was a significant difference in mean constant coefficient for TOC for north and south of Limes Norrlandicus, (0.83 and 1.8, respectively). The TOC constant coefficient had higher correlations than AbsF_mgPtL_ constant coefficients for north of Limes Norrlandicus and for the smallest and largest catchments than for the Fe constant coefficient. For the second largest catchments and south of Limes Norrlandicus, the Fe constant and TOC constant correlations with the AbsF_mgPtL_ constant coefficient were similar. For the second smallest catchments, the Fe constant had higher correlations than the TOC constant with the AbsF_mgPtL_ constant coefficient. The forest-dominated catchments had significantly higher (1 − *β* > 0.80) mean constant coefficients for all four water quality parameters compared to the other land classes, except for the mean Fe constant in forest- and wetland-dominated catchments (Fig. S2, Electronic Supplementary Material). This, despite forest-dominated catchments having significantly higher mean, measured AbsF_mgPtL_, AbsUnF_mgPtL_, and TOC than mixed land use catchments.

### The amplitude, the impact from the seasonality on the water quality parameters

The seasonality (amplitude) of the 99 watercourses with significant log-linear models had the lowest impact on TOC concentration compared with the other three water quality parameters (Table 2). The mean amplitudes for AbsF_mgPtL_, AbsUnF_mgPtL_, Fe, and TOC were 0.24, 0.25, 0.30, and 0.18, respectively. The amplitudes of Fe and AbsUnF_mgPtL_ were significantly higher (1 − *β* > 0.80) than the amplitude of TOC. In larger catchments (>725 km^2^), the impact of seasonality on TOC and Fe (1 − *β* > 0.80) differed more compared to the smaller catchments that had 1 − *β* < 0.6. Correlations between the amplitudes of AbsF_mgPtL_ and Fe or AbsF_mgPtL_ and TOC were higher for TOC for all area classes and for both north and south of Limes Norrlandicus.

**Fig. 2:**
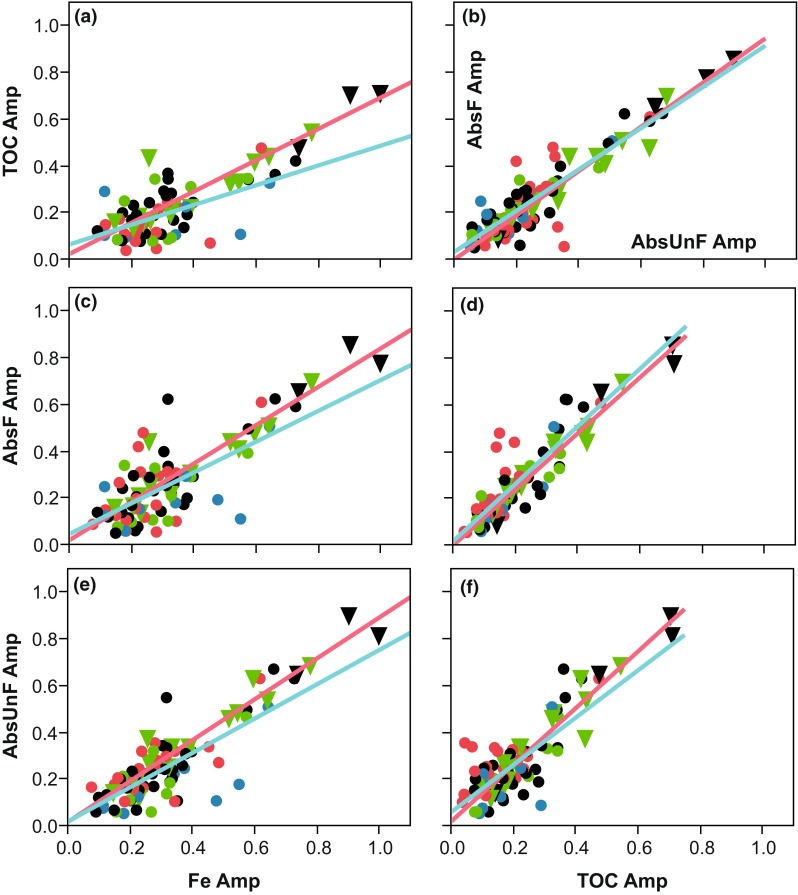
Correlations between Fluxmaster calculated significant (*P* < 0.05) amplitude for AbsF_mgPtL_, AbsUnF_mgPtL_, Fe, and TOC. The *colors* on the *dots* in the graph represent land use (*red* for agriculture, *green* for forest, *blue* for wetland and *black* for mixed land use), and the shape represents pH concentration (triangle: pH < 6 and dots: 6 > pH). Red line is regression based on data with pH < 6, *blue line* is 6 > pH

The highest *r* ^2^-value obtained by the weighted linear regressions was between AbsF_mgPtL_ and TOC (0.87, see Fig. 2d). When the amplitude for Fe was involved, the *r* ^2^-value in the weighted linear regression was lower, around 0.6 for AbsF_mgPtL_ and TOC (Fig. 2a, c). For the regression between Fe and AbsUnF_mgPtL_, the *r* ^2^-value was higher, 0.77 (Table 3). At low amplitudes, more scatter can be observed. This was especially true for the correlations between the amplitude of Fe versus TOC and Fe versus AbsF_mgPtL_ (Fig. 2a and c, respectively) that revealed scatter at low values, where agricultural areas predominate. On the other hand, catchments with high amplitudes tended to show strong correlations for all parameters. The two pH-classes (median pH below or above 6.0; Table 1) gave significant pH-class influence on amplitude for TOC versus AbsF_mgPtL_, TOC versus Fe, and AbsF_mgPtL_ versus AbsUnF_mgPtL_ (Table 3; Fig. 2a, b, d), decreasing respective amplitudes at higher pH. The sulfate and alkalinity classes did not give any significant contributions to the weighted linear regressions.

**Table 3:** Weighted linear regression between Fluxmaster calculated amplitudes. The regressions were weighted by the sum of *r* ^2^ for the two parameters involved. No constants were significant and for some regressions the pH-classes made a significant change in the regression for watercourses with median pH < 6.0 or pH > 6.0

Var	Coefficients	Estimate	SE	*t* value	Pr(>|*t*|)	Adjusted *R* ^2^	*p* value	*F* statistic
TOC Amp	AbsF Amp	0.954	0.11	8.53	<0.001	0.90	<0.001	289
TOC Amp	AbsF Amp + class pH	−0.140	0.06	−2.17	0.03			
AbsUnF Amp	AbsF Amp	1.203	0.16	7.70	<0.001	0.87	<0.001	211
AbsUnF Amp	AbsF Amp + class pH	−0.196	0.09	−2.17	0.03			
TOC Amp	AbsUnF Amp	0.930	0.15	6.28	<0.001	0.80	<0.001	135
AbsUnF Amp	Fe Amp	0.983	0.17	5.86	<0.001	0.78	<0.001	117
AbsF Amp	Fe Amp	0.993	0.21	4.77	<0.001	0.67	<0.001	67
TOC Amp	Fe Amp	0.900	0.17	5.45	<0.001	0.67	<0.001	68
TOC Amp	Fe Amp + class pH	−0.244	0.10	−2.43	0.02			

**Fig. 3:**
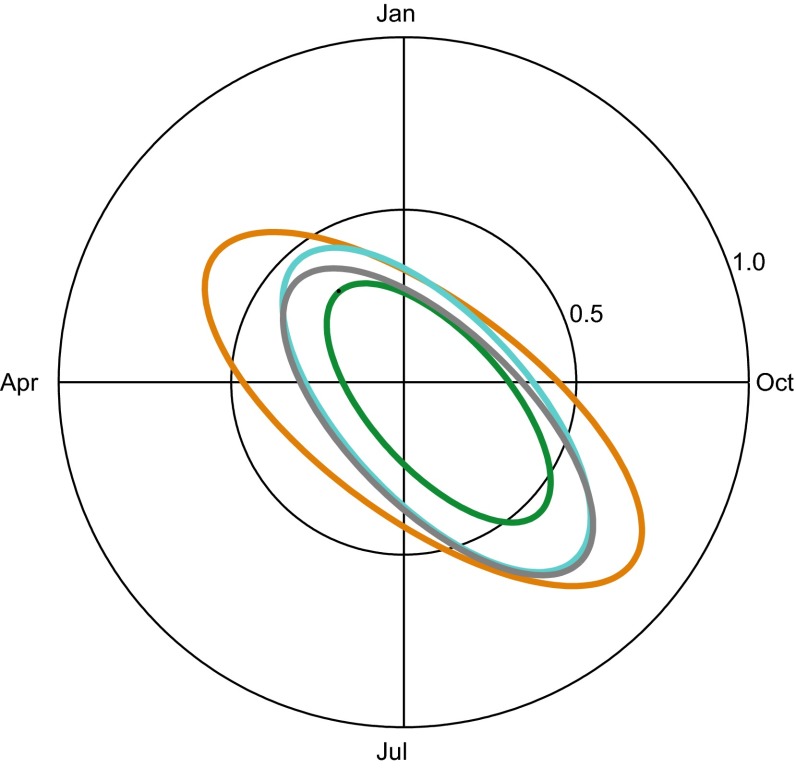
Year plot showing 95 % elliptic confidence intervals of amplitude and peak day from the 99 watercourses with significant Fluxmaster models. The AbsF_mgPtL_ (*turquoise*), AbsUnF_mgPtL_ (*gray*), Fe (*orange*), and TOC ellipse in (*green*)

The peaks in the seasonality terms usually occurred in August and September or, for a few watercourses 6 months later in February–March (Fig. 3). The seasonality peak for Fe often lagged behind the peaks for the other three water quality parameters in both fall and spring (Fig. S3, Electronic Supplementary Material). Fe had large difference between median and mean peak day, 204 and 177 Julian days, respectively (Table 2). In Fig. 3, the 95 % confidence interval ellipse indicates that Fe seasonality was more dispersed between watercourses than the other water quality parameters with more peak days during the spring and with higher amplitudes than the other three water quality parameters. For TOC, the watercourses behaved more synchronously, with lower amplitudes and similar occurrence of peak day in August–September. AbsF_mgPtL_ and AbsUnF_mgPtL_ have nearly identical ellipses. Correlations between AbsF_mgPtL_ peak day and peak day of Fe or TOC were higher for TOC, for both north and south of Limes Norrlandicus and for largest catchments (>725 km^2^). For smallest catchments (<20 km^2^), Fe peak day had higher correlations with AbsF_mgPtL_ than TOC had, while for second smallest catchments (21–624 km^2^), the correlations were rather similar.

### Discharge dependency of the water quality parameters

**Fig. 4:**
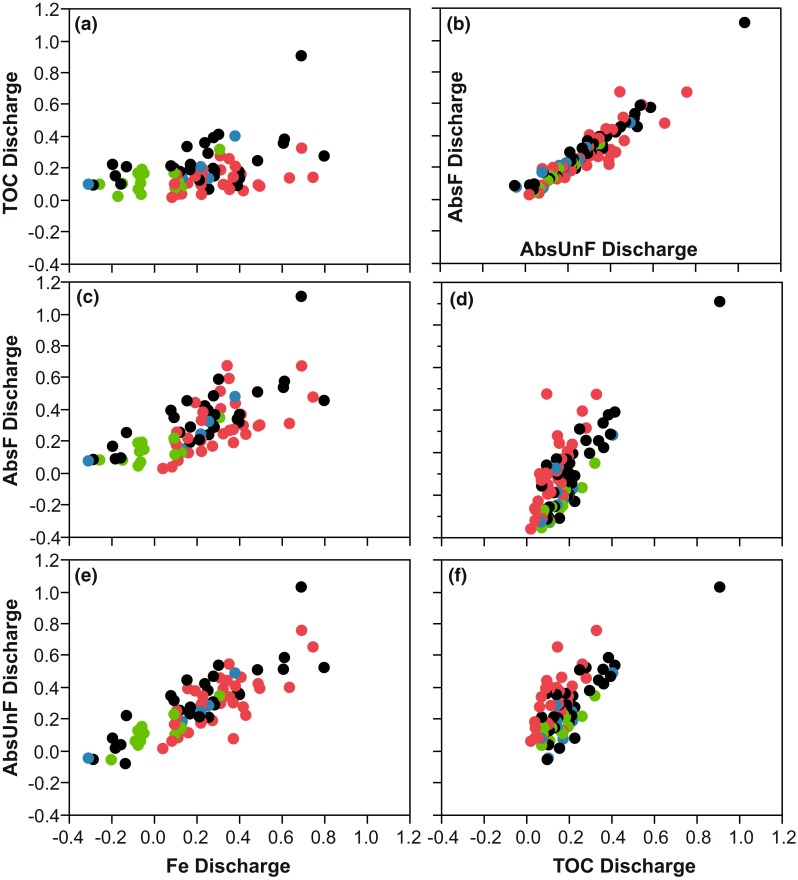
Correlations between Fluxmaster calculated significant (*P* < 0.05) Discharge coefficient for AbsF_mgPtL_, AbsUnF_mgPtL_, Fe, and TOC. The colors on the dots in the graph represent land use (*red* for agriculture, *green* for forest, *blue* for wetland, and *black* for mixed land use)

TOC and Fe had similar mean discharge coefficients (0.15), which were 1 − *β* > 0.80 significantly lower than both those for AbsF_mgPtL_ (0.25) and AbsUnF_mgPtL_ (0.23), based on the 99 watercourses with significant log-linear models. The discharge coefficients for Fe and TOC did not correlate very well (Fig. 4a). The TOCs’ discharge coefficients are low, except for one outlier, and two negative coefficients. Of all catchments, 29 watercourses had negative Fe discharge coefficients and a greater amount of variability than the TOCs discharge coefficients. For the smallest catchments, (<20 km^2^) Fe had a significantly smaller mean discharge coefficient than the others water quality parameters. In the second largest catchments (725–3442 km^2^), the difference in mean discharge coefficients for TOC was significantly (1 − *β* > 0.95) lower than those for AbsF_mgPtL_ and AbsUnF_mgPtL_.

In the regression plots of discharge coefficients between TOC and AbsF_mgPtL_ as well as TOC and AbsUnF_mgPtL_, the watercourses with a large agricultural percentage (>5 %) were almost all above the regression line, while the opposite was true for the forest catchments (Fig. 4d, f). Correlations between the discharge coefficients for AbsF_mgPtL_ and TOC were slightly higher for those for discharge coefficients associated with TOC and Fe, for all area classes and for both north and south of Limes Norrlandicus. AbsF_mgPtL_, AbsUnF_mgPtL_, and Fe were significantly (1 − *β* > 0.80) more controlled by discharge in agricultural or mixed land use areas than in forest-dominated catchments. There was no significant difference in the mean discharge coefficient for TOC between the land classes. Catchments for south of Limes Norrlandicus had significantly (1 − *β* > 0.80) higher mean AbsF_mgPtL_ and AbsUnF_mgPtL_ discharge coefficients compared to those for TOC. Catchments for north of Limes Norrlandicus had significantly higher discharge coefficients for Fe than for AbsF_mgPtL_.

### Long-term changes in water quality parameters concentration over time

**Fig. 5:**
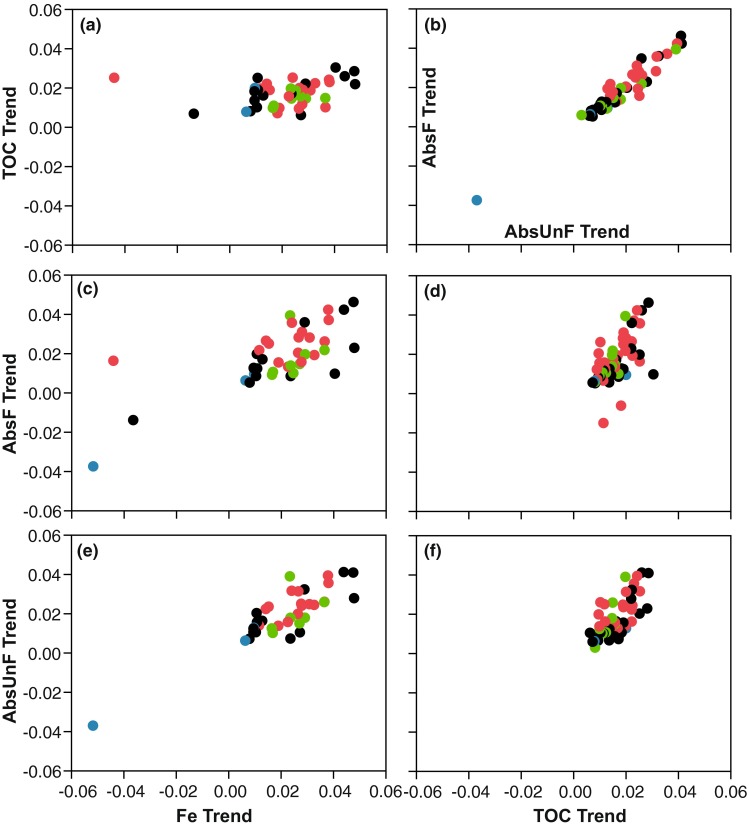
Correlations between Fluxmaster calculated significant (*P* < 0.05) trends for AbsF_mgPtL_, AbsUnF_mgPtL_, Fe, and TOC. The *colors* on the *dots* in the graph represent land use (*red* for agriculture, *green* for forest, *blue* for wetland, and *black* for mixed land use)

The mean trend coefficient in the 99 watercourses with significant log-linear models did not differ significantly between the four water quality parameters, 0.011 for TOC, 0.0085 for Fe, 0.0099 for AbsF_mgPtL_, and 0.0098 for AbsUnF_mgPtL_. While the mean values did not differ, correlations between parameters did vary (Fig. 5). There were more negative Fe trend coefficients (*n* = 17) than for the other three water quality parameters. In the southern part of Sweden, the trend coefficient was significantly higher (1 − *β* > 0.97) for AbsF_mgPtL_ and AbsUnF_mgPtL_ with values of 0.0048 (southern) versus 0.015 (northern) and 0.0053 versus 0.014, respectively. Mean TOC trend coefficients were significantly higher (1 − *β* of 0.93) in the second largest catchments (0.015) and largest (0.014) compared to the smallest catchments (0.0059). The other three water quality parameters did not have significant changes in mean trend coefficients with area classes.

### Factor analysis (FA)

**Fig. 6:**
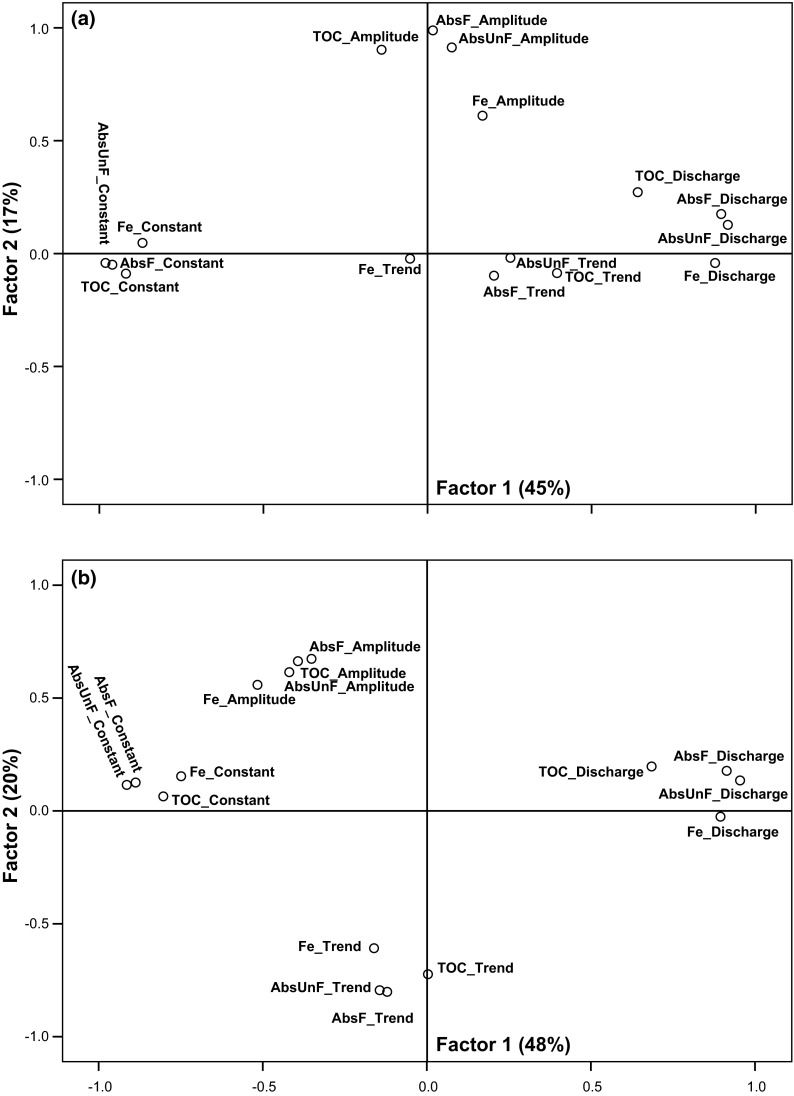
Factor analysis on 99 watercourses with significant Fluxmaster models for all four water quality parameters. Where **a** is watercourses north of Limes Norrlandicus and **b** is south thereof

All shown FAs were valid to perform since KMO >0.70 and Bartlett’s test of sphericity *P* values was less than <0.001 (see Electronic Supplementary Material). The 99 watercourses with significant log-linear models displayed similar patterns of Fluxmaster coefficients for all four water quality parameters in the FA. In south of Limes Norrlandicus, similar pattern was seen for the smallest catchments (<624 km^2^) (Fig. 6). The Fe trend coefficient showed a different behavior for north of Limes Norrlandicus; it was close to origin and not close to the other water qualities. The largest catchments (>725 km^2^) were not valid for performing FA. PCA did not capture this difference of Fe north of Limes Norrlandicus (data not shown) but showed a pattern similar to that from FA south of the border.

### PLS

None of the four water quality parameters coefficients were well modeled by PLS with the available explanatory data (Table 4). The highest *Q* ^2^ (see Electronic Supplementary Material) in the PLS-modeling of Fluxmaster coefficients for individual water quality parameters was obtained for AbsF_mgPtL_ (median *Q* ^2^ of 0.25) and the lowest for AbsUnF_mgPtL_ (*Q* ^2^ = 0.13). For the log-linear model coefficients, PLS-models had highest *Q* ^2^ for the constant coefficient (median *Q* ^2^ of 0.71), while the modeling power for the other terms was low (Table 4).

**Table 4:** PLS results per water quality parameters, Fluxmaster coefficients, and modeling performances

Term	*R* ^2^	*Q* ^2^
AbsF	0.31	0.25
AbsUnF	0.14	0.13
Fe	0.27	0.23
TOC	0.21	0.15
Constant	0.73	0.71
Discharge	0.38	0.35
Trend	0.27	0.21
Amplitude	0.39	0.34
*r* ^2^	0.12	0.08
NSE	0.15	0.10

PLS-modeling of the 99 watercourses with significant log-linear models using one Fluxmaster coefficient at a time for all four water quality parameters performed well for the constant and discharge coefficients when comparing catchments for south and north of Limes Norrlandicus. For the trend and amplitude coefficients, *Q* ^2^ > 0.2 was obtained for south of the Limes Norrlandicus, while north thereof, *Q* ^2^ was below zero.

Catchment size always had negative coefficients (VIP >1) (see Electronic Supplementary Material), for all four water quality parameters and log-linear terms except for the trend coefficient. Higher lake surface coverage (%) always gave lower concentrations and lower values for amplitude and a lower *r* ^2^.

## Discussion

Time series analysis such as the one conducted here is one of the major uses of monitoring data. The Fluxmaster approach has several advantages as compared to site-specific trend analysis: (i) It may reveal seasonality and discharge driven effects that otherwise are hidden while still delivering information about long-term trends. (ii) The post hoc analysis of differing effects of catchments types on seasonality, trend, and discharge driven factors may reveal important spatial and landscape type patterns. (iii) The chemical parameters AbsF, Fe, and TOC could be fitted to a log-linear model at almost all catchments.

The close co-variation of factors and trends is not surprising given common source areas in the near-stream zone and common mobilization mechanisms at varying discharge and similar influences of vegetation. Nevertheless, some distinct differences that are connected to specific landscape characteristic, slight differences in mobilization, and varying degree of processing downstream are discussed in detail below. In general, watercourses dominated by forests had higher amplitude values and lower discharge coefficient values than watercourses in agricultural catchments. The seasonality peak for Fe often lagged behind the peaks for the other three water quality parameters in both fall and spring peak catchments (Fig. S3, Electronic Supplementary Material). For the smallest catchments (0.2–20 km^2^), Fe peak day had higher correlations with AbsF_mgPtL_ than TOC had, while for the second smallest catchments, (21–624 km^2^), the correlations were rather similar. From this, it can be concluded that at lower pH in the smallest catchments, Fe has a bigger effect on AbsF. In the larger catchments, (>725 km^2^) more than 50 % of the Fe is particulate. However, other metals such as manganese (Mn) could also influence AbsF.

This co-variation (prediction 1) may be explained by similar mobilization mechanisms that are related to discharge and seasonality (prediction 2). Longer water retention time will allow for selective removal processes (Köhler et al. 2013), dampening, and weakening the co-variation (prediction 3). The relatively poor performance of models for Fe could be due to the mobilization of Fe together with soil or mineral particles (Björkvald et al. 2008) and the transformation of iron from the dissolved to the particulate phase as pH increases. Fe is known to form colloidal associations with organic matter that will pass a 0.45 µm filter. Recently, Neubauer et al. (2013) presented evidence that Fe-particulates will be increasingly removed in a 0.45 µm filter as solution pH increases, retaining as much as 50 % when pH is above 6. We would thus expect a better correlation between AbsF_mgPtL_ and Fe at pH below pH 6 as compared to catchments with pH above 6 (Table 3). The log-linear (Fluxmaster) model presented here did not use pH and that could be one reason why the model worked better for the other three water quality parameters.

Meili (1992) observed no correlation between the color/TOC-ratio and pH in 18 Swedish forest-dominated humic soft water lakes, although more recent work links the increasing color/TOC-ratios to increased pH (SanClements et al. 2012). Erlandsson et al. (2012), however, reported that lake color is negatively affected by high buffering capacity (high pH) and high percentage land cover of agriculture in the catchment. This observation is in accordance with the low amplitudes of AbsF models that have been observed in the agriculture-dominated areas in this study.

The strong correlations between AbsF and AbsUnF across scales were somewhat unexpected at first sight. While AbsF is included in the measurement of AbsUnF, the dataset includes many larger rivers and streams including agricultural systems where the presence of particles should lead to larger differences. An analysis of the long-term median values at all catchments revealed that AbsF and AbsUnF did not differ drastically. In areas with pH below 6, AbsF (0.31) and AbsUnF (0.37) mean values differ little (1 − *β* of 0.35), and the average slope was 0.9. In areas with pH above 6, this slope was 0.7 (mean AbsF of 0.15 and AbsUnF of 0.22 with 1 − *β* of 0.62). For the agricultural areas, the slope was 0.6 while it was 0.8 for all other areas with pH >6. These significant but small differences in the more alkaline waters indicate that AbsUnF is probably not a good measure for the presence of mineral particles. Many authors have proposed that the presence of Fe affects water color (Eriksson 1929; Åberg and Rodhe 1942; Kritzberg and Ekström 2012; Köhler et al. 2013). Köhler et al. (2013) have shown that the average contribution of dissolved Fe to AbsF is around 40 %. The close correlation of AbsF and AbsUnF could indicate that different Fe forms were an important driver of the observed patterns. High alkaline waters would tend to loose AbsF_mgPtL_ and form AbsUnF_mgPtL_ when Fe forms particulate Fe. These particles, however, seem though to be too small to be effectively removed from the aqueous phase. As a result, AbsF_mgPtL_ and AbsUnF_mgPtL_ co-vary.

Multiple regression models between the long-term median values for Fe, TOC, and AbsUnF_mgPtL_ reveal that Fe and TOC could explain more than 87 % (agricultural dominating catchments), 94 % (forest) and up to 98 % (wetland and mixture) of the observed variations in AbsUnF_mgPtL_. This is consistent with what is known of organic matter-mediated Fe mobilization in the landscape (Dillon and Molot 1997; Björkvald et al. 2008). For the agricultural catchments, the contribution of Fe, in the multiple regressions, was twice as large as that of the forests or wetlands. We may thus conclude that the contribution of Fe-rich particles in the agricultural areas was much larger than at all other catchments. The presence of these particles in the agricultural-dominated watercourses was most probably the cause for the poor correlations of Fe with the other three water quality parameters. A large part of the agricultural catchments are located in the South Swedish plains where silt and clay soils are the dominating soil type. The larger trend in AbsUnF in the southern part of Sweden could be due to an increase of particulate Fe from the agricultural areas. This rise in turbidity is potentially of concern for aquatic organisms in those watercourses as turbidity is known to strongly affect occurrence of macrophytes and the competition between different cyanobacteria species (Bilotta and Brazier 2008).

Kritzberg and Ekström (2012) analyzed monitoring data from 1972 to 2010 for 30 rivers draining to the Swedish coast. By comparing the effect of experimental Fe additions on water color with the variation in water color and Fe concentration in the monitoring data, they showed that Fe can explain a significant share of the variation in water color (on average 25 %), especially in the rivers in the north of Sweden (up to 74 %). Furthermore, positive trends for iron were seen in 27 of 30 rivers (21–468 %), and the increase in Fe was larger than that of organic matter. This indicates that Fe and organic matter concentrations are controlled by similar but not identical processes (Dillon and Molot 2005; Kortelainen et al. 2006; Neal et al. 2008; Kritzberg and Ekström 2012; Sarkkola et al. 2013).

The seasonality term can be considered as a surrogate for soil temperature, and it is likely that soil temperature can replace the seasonality term (Hytteborn et al. unpublished results), so watercourses with high amplitude are likely to be more sensitive to soil temperature. High amplitudes tend to coincide spatially (Fig. 2). Most agricultural catchments tend to have lower co-varying amplitudes for AbsF_mgPtL_, AbsUnF_mgPtL_, and TOC, while the Fe amplitude can be higher or lower. The wetland catchments, on the other hand, tend to have higher amplitudes for Fe than the other three water quality parameters. This could be due to redox driven processes that are not related to seasonality (Dillon and Molot 1997). Both Dillon and Molot (1997) and Björkvald et al. (2008) have shown that wetland dominated areas have a three to four times larger annual variation in Fe concentrations as compared to forest catchments. The mean amplitudes for Fe and TOC differed significantly (1 − *β* > 0.80) between agriculture-dominated catchments (Fe = 0.12 and TOC = 0.21) and those dominated by forests (Fe = 0.24 and TOC = 0.35).

The trend-coefficient was significantly (1 − *β* > 0.95) lower in the north for both AbsF_mgPtL_ and AbsUnF_mgPtL_ than it was in the south. We expect that an increase of high discharge events in the south might thus lead to an increase in color and more variable water color, while effects of TOC that often are of primary concern for drinking water will probably be less pronounced. AbsF_mgPtL_ and AbsUnF_mgPtL_ trend terms were significantly higher in agricultural-dominated catchments than those dominated by wetlands. An increase in color per TOC is connected to more terrestrial C (Haaland et al. 2010; SanClements et al. 2012; Köhler et al. 2013) but in the drinking water facilities probably more easily removed by flocculation (Kothawala et al. 2014).

A small difference (indicated by FA but no significant difference in mean) in Fe trend was observed between north and south, despite the significant difference in mean AbsUnF_mgPtL_. Agriculture-dominated catchments had significantly higher TOC mean trends than those dominated by forests; this could be the reason why the largest catchments (>725 km^2^) had larger trends than the smallest catchments (<20 km^2^). The underlying reasons for this observation are not known, but we can speculate that recovery from acid rain and a rising primary production due to warmer temperature (Larsen et al. 2011) and ongoing nitrogen deposition may play a major role.

While lakes of moderate to large volumes upstream of the sampling point decrease the concentration of the four water quality parameters (Eriksson 1929), the impact is larger for Fe than for TOC (Weyhenmeyer et al. 2014). However, Weyhenmeyer et al. (2014) used data from 6339 lakes (and watercourses), different time periods and different means of evaluating the data. They included other water chemistry, pH, and silicate (Si), in their PLS-modeling of AbsF, but they did not include the effect of pH on dissolved or particulate Fe.

This study has demonstrated that empirical nonlinear modeling of long time series in combination with powerful statistics is a valuable tool for deciphering nonlinear trends and landscape features on a countrywide scale. The observed catchment specific responses of land use, catchment size, change over time (trend), and response to discharge will allow to make some prediction of future changes in water quality that are relevant for a number of interest groups such as local authorities, fishery boards or drinking water suppliers.

Understanding water color, Fe, and TOC, seasonal dynamics and their relationship to discharge may provide important insights into current climatic controls on water quality and hence the possibility of browner water in the future

## Conclusion

Our results show that while there is some synchrony in the behavior of water quality parameters associated with surface water browning, iron, TOC, and water color all respond to flow, albeit in slightly different ways. For example, seasonality peaks for iron often lagged behind those for the other three water quality parameters. The importance of lakes as a driver in spatial and temporal patterns of iron, TOC, and water color was apparent; a greater percentage of lake surface area in the catchment always gave lower concentrations, a smaller seasonal signal, and more unexplained variability. In agricultural areas, the contribution of iron, in multiple regression models, was twice as large as that of the forests or wetlands. Larger catchments, which usually co-vary with more agricultural lands, had significant larger trends (of iron, TOC and water color) than small forest catchments. Agricultural land use impacts both Fe and color patterns. Long-term trends in all parameters are consistent with a recovery from acidification. Regression models similar to those presented here can be a useful tool for understanding similarities and differences in the behavior of water quality parameters associated with browning. This understanding is necessary for the future management of drinking water resources and understanding the consequences of global change on aquatic ecosystems in the boreal landscape.

## Electronic supplementary material

Below is the link to the electronic supplementary material.

Supplementary material 1 (PDF 770 kb)

## References

[CR1] Åberg B, Rodhe W (1942). Über die Milieufaktoren in einigen Südschwedischen Seen.

[CR2] Bilotta GS, Brazier RE (2008). Understanding the influence of suspended solids on water quality and aquatic biota. Water Research.

[CR3] Björkvald L, Buffam I, Laudon H, Mörth C-M (2008). Hydrogeochemistry of Fe and Mn in small boreal streams: The role of seasonality, landscape type and scale. Geochimica et Cosmochimica Acta.

[CR4] Bossard, M., J. Feranec, and J. Otahel. 2000. CORINE land cover technical guide—Addendum 2000, European Environment Agency, Copenhagen, 105 pp. Retrieved, from http://www.eea.europa.eu/publications/COR0-landcover. Accessed 8 Nov 2012.

[CR47] Cohn, T.A., D.L. Caulder, E.J. Gilroy, L.D. Zynjuk, and R.M. Summers. 1992. The validity of a simple statistical model for estimating fluvial constituent loads: An empirical study involving nutrient loads entering Chesapeake Bay.* Water Resources Research* 28: 2353–2363. doi:10.1029/92wr01008.

[CR5] De’ath, G., and K.E. Fabricius. 2000. Classification and regression trees: A powerful yet simple technique for ecological data analysis. *Ecology* 81: 3178–3192. doi:10.1890/0012-9658(2000)081%5B3178:CARTAP%5D2.0.CO;2.

[CR6] Dillon PJ, Molot LA (1997). Effect of landscape form on export of dissolved organic carbon, iron, and phosphorus from forested stream catchments. Water Resources Research.

[CR7] Dillon PJ, Molot LA (2005). Long-term trends in catchment export and lake retention of dissolved organic carbon, dissolved organic nitrogen, total iron, and total phosphorus: The Dorset, Ontario, study, 1978–1998. Journal of Geophysical Research.

[CR8] Eriksson, J.V. 1929. Den kemiska denudationen i Sverige (The chemical denudation of Sweden). Report Band 5, nr 3, Swedish Meteorological and Hydrological Institute, Stockholm, 95 pp. (In Swedish with French summary).

[CR9] Erlandsson M, Futter MN, Kothawala DN, Köhler SJ (2012). Variability in spectral absorbance metrics across boreal lake waters. Journal of Environmental Monitoring.

[CR10] Fölster, J. R.K. Johnson, M.N. Futter, and A. Wilander. 2014. The Swedish monitoring of surface waters: 50 years of adaptive monitoring. *AMBIO.* doi:10.1007/s13280-014-0558-z. 10.1007/s13280-014-0558-z PMC4235935 25403966

[CR11] Forsberg C, Petersen P (1990). A darkening of Swedish lakes due to increased humus inputs during the last 15 years. Verhandlungen Internationale Vereingung für theoretische und angewandte Limnologie.

[CR12] Gadmar TC, Vogt RD, Østerhus B (2002). The merits of the high temperature combustion method for determining the amount of natural organic carbon in surface freshwater samples. International Journal of Environmental Analytical Chemistry.

[CR13] Haaland S, Hongve D, Laudon H, Riise G, Vogt RD (2010). Quantifying the drivers of the increasing colored organic matter in boreal surface waters. Environmental Science and Technology.

[CR14] Ivarsson H, Jansson M (1994). Temporal variations in the concentration and character of dissolved organic matter in a highly colored stream in the coastal zone of northern Sweden. Archiv für Hydrobiologie.

[CR15] Johansson, H. 2002. On the distribution on coefficients in aquatic systems. PhD Thesis. Uppsala University, Uppsala.

[CR16] Karlsson J, Byström P, Ask J, Ask P, Persson L, Jansson M (2009). Light limitation of nutrient-poor lake ecosystems. Nature.

[CR17] Knorr KH (2013). DOC-dynamics in a small headwater catchment as driven by redox fluctuations and hydrological flow paths—Are DOC exports mediated by iron reduction/oxidation cycles?. Biogeosciences.

[CR18] Köhler, S. 1999. Quantifying the role of natural organic acids on pH and buffering in Swedish surface waters. PhD Thesis, Swedish University of Agricultural Sciences, Umeå.

[CR19] Köhler SJ, Kothawala D, Futter MN, Liungman O, Tranvik L (2013). In-lake processes offset increased terrestrial inputs of dissolved organic carbon and color to lakes. PLoS One.

[CR20] Kortelainen P, Mattsson T, Finer L, Ahtiainen M, Saukkonen S, Sallantaus T (2006). Controls on the export of C, N, P and Fe from undisturbed boreal catchments, Finland. Aquatic Sciences.

[CR21] Kothawala DN, Stedmon CA, Müller RA, Weyhenmeyer GA, Köhler SJ, Tranvik LJ (2014). Controls of dissolved organic matter quality: Evidence from a large-scale boreal lake survey. Global Change Biology.

[CR22] Kritzberg ES, Ekström SM (2012). Increasing iron concentrations in surface waters—A factor behind brownification?. Biogeosciences.

[CR23] Larsen S, Andersen TOM, Hessen DO (2011). Climate change predicted to cause severe increase of organic carbon in lakes. Global Change Biology.

[CR24] Lindström G, Pers C, Rosberg J, Strömqvist J, Arheimer B (2010). Development and testing of the HYPE (Hydrological Predictions for the Environment) water quality model for different spatial scales. Hydrology Research.

[CR25] Meili M (1992). Sources, concentrations and characteristics of organic matter in softwater lakes and streams of the Swedish forest region. Hydrobiologia.

[CR26] Monteith DT, Stoddard JL, Evans CD, de Wit HA, Forsius M, Hogasen T, Wilander A, Skjelkvåle BL (2007). Dissolved organic carbon trends resulting from changes in atmospheric deposition chemistry. Nature.

[CR27] Müller RA, Futter MN, Sobek S, Nisell J, Bishop K, Weyhenmeyer GA (2013). Water renewal along the aquatic continuum offsets cumulative retention by lakes: Implications for the character of organic carbon in boreal lakes. Aquatic Sciences.

[CR28] Nash JE, Sutcliffe JV (1970). River flow forecasting through conceptual models part I—A discussion of principles. Journal of Hydrology.

[CR29] Neal C, Lofts S, Evans CD, Reynolds B, Tipping E, Neal M (2008). Increasing iron concentrations in UK upland waters. Aquatic Geochemistry.

[CR30] Neubauer E, Köhler SJ, von der Kammer F, Laudon H, Hofmann T (2013). Effect of pH and stream order on iron and arsenic speciation in boreal catchments. Environmental Science and Technology.

[CR31] Reese, H., M. Nilsson, T. Granqvist Pahlén, O. Hagner, S. Joyce, U. Tingelöf, M. Egberth, and H. Olsson. 2003. Countrywide estimates of forest variables using satellite data and field data from the national forest inventory. *AMBIO* 33: 542–548. doi:10.1579/0044-7447-32.8.542. 10.1579/0044-7447-32.8.542 15049351

[CR32] Roulet N, Moore TR (2006). Browning the waters. Nature.

[CR33] SanClements MD, Oelsner GP, McKnight DM, Stoddard JL, Nelson SJ (2012). New insights into the source of decadal increases of dissolved organic matter in acid-sensitive lakes of the Northeastern United States. Environmental Science and Technology.

[CR34] Sarkkola S, Nieminen M, Koivusalo H, Laurén A, Kortelainen P, Mattsson T, Palviainen M, Piirainen S (2013). Iron concentrations are increasing in surface waters from forested headwater catchments in eastern Finland. Science of the Total Environment.

[CR35] Schwarz, G.E., A.B. Hoos, R.B. Alexander, and R.A. Smith. 2006. The SPARROW surface water-quality model: Theory, application, and user documentation. U.S. Geological Survey Techniques and Methods Report, Book 6, Chapter B3, U.S. Geological Survey, 248 pp. Retrieved, from http://pubs.usgs.gov/tm/2006/tm6b3/PDF.htm. Accessed 10 Dec 2011.

[CR36] SEPA. 2007. *Kartläggning och analys av ytvatten (Identification and analysis of surface water, in Swedish). Handbook 2007:3*. Stockholm: Swedish Environmental Protection Agency, 81 pp. http://www.naturvardsverket.se/Documents/publikationer/620-0146-9.pdf. Accessed 11 Dec 2013.

[CR37] Sernander, R. 1901. *Den skandinaviska vegetationens spridningsbiologi*. Berlin: Friedländer. (In Swedish with a German summary).

[CR38] Sjöstedt CS, Gustafsson JP, Köhler SJ (2010). Chemical equilibrium modeling of organic acids, pH, aluminum, and iron in Swedish surface waters. Environmental Science and Technology.

[CR39] SLU. 2011. Water chemical analysis. Retrieved 10 November, 2011, from http://www.slu.se/en/faculties/nl/about-the-faculty/departments/department-of-aquatic-sciences-and-assessment/laboratories/geochemical-laboratory/water-chemical-analyses/.

[CR40] Snucins E, Gunn J (2000). Interannual variation in the thermal structure of clear and colored lakes. Limnology and Oceanography.

[CR41] Spellman FR, Drinan JE (2012). The drinking water handbook.

[CR42] Vuorenmaa J, Forsius M, Mannio J (2006). Increasing trends of total organic carbon concentrations in small forest lakes in Finland from 1987 to 2003. Science of the Total Environment.

[CR43] Wallace JB, Eggert SL, Meyer JL, Webster JR (1997). Multiple trophic levels of a forest stream linked to terrestrial litter inputs. Science.

[CR44] Weyhenmeyer GA, Fröberg M, Karltun E, Khalili M, Kothawala D, Temnerud J, Tranvik LJ (2012). Selective decay of terrestrial organic carbon during transport from land to sea. Global Change Biology.

[CR45] Weyhenmeyer GA, Prairie YT, Tranvik LJ (2014). Browning of boreal freshwaters coupled to carbon-iron interactions along the aquatic continuum. PLoS One.

