# Supplementary material for: Evaluating common drivers for color, iron and organic carbon in Swedish watercourses
Source: Ambio. 2014 Nov 15;43(Suppl 1):30–44. doi: 10.1007/s13280-014-0560-5 (PMC4235929; doi:10.1007/s13280-014-0560-5)
Supplement: Supplementary file 1 — Supplementary material 1 (PDF 770 kb) [file 13280_2014_560_MOESM1_ESM.pdf]

AMBIO

Electronic Supplementary Material

Evaluating common drivers for color, iron and organic carbon in Swedish watercourses
This supplementary material has not been peer reviewed.

Johan Temnerud, Julia Kristina Hytteborn, Martyn Norman Futter, Stephan Jürgen Köhler

Supplementary Material

Statistical Methods

Statistical Power Analysis

Statistical power analysis was performed using G*Power 3.1 (Faul et al. 2009). The post hoc power analysis of the difference in means between groups used a two-tailed Student t-test to evaluate the H0 that there is no difference between the groups. H1 was that there is a difference between groups. The statistical power (1-β) gives the probability that the hypothesis is correctly rejected; a value above 0.80 indicates that H0 was correctly rejected. The effect size (Cohen's d) is the absolute value of (x1-x2)/(pooled standard deviation of x1 & x2) (Cohen 1988). Where x1 and x2 are one of the different groups or classes; north and south of Limes Norrlandicus, catchment size classes and dominant land use in the catchment. Pearson and partial correlations and Spearman ranking were calculated on the coefficients from the log-linear models.

Common factor analysis (FA)

Common factor analysis (FA), also called principal axis factoring, was performed using the oblique (non-orthogonal) rotation method Oblimin (d = 0) and Kaiser normalization (using SPSS v21). The Kaiser-Meyer-Olkin (KMO) measure of sampling adequacy tests whether the partial correlations among variables are small enough to ensure the validity of the FA. The KMO should be greater than 0.6 for a FA to be valid. Another indicator of the strength of the relationship among variables is Bartlett's test of sphericity, which is used to test the null hypothesis that the variables in the correlation matrix are uncorrelated. When the significance level is small enough to reject the hypothesis ($p < 0.0001$), the relationship among variables is strong, and FA can be conducted. In FA the

variance of a single variable is decomposed into common variance that is shared by other variables included in the model and unique variance that is unique to a particular variable (Gauch 1982). FA is interpreted in a similar manner to principal component analysis (PCA), with the difference that PCA considers only the total variance and makes no distinction between common and unique variance whereas FA does (Gauch 1982). Oblique rotation allows in FA facilitates correlate amongst factors. If the factors are truly uncorrelated, orthogonal and oblique rotation produces similar results (Costello and Osborne 2005).

Partial least squares regression (PLS)

Partial least squares regression (PLS) was used to explore if catchment characteristics could predict the Fluxmaster coefficients (constant, amplitude, discharge and trend) for the watercourses that had significant Fluxmaster models. All data, both Fluxmaster coefficients and catchment characteristics, were centered by mean normalization and weighted by dividing the variables with the standard deviation prior to PLS in SIMCA for Windows v13.0 (Umetrics). PLS identifies the relationship between independent and dependent data matrices through a linear, multivariate model and is less sensitive to autocorrelated, independent variables when compared to multiple linear regression approaches (Geladi and Kowalski 1986). Variables were $\log_{10}(X+C)$ transformed to better approximate a normal distribution if any of the following criteria were fulfilled: 1. $0 \le Min(X)/Max(X) \le 0.1$ or 2. $S < -2$ or $S > 2$, where $S = Skewness/\sqrt{(6n(n-1)/((n-2)(n+1)(n+3)))}$. X is the variable, n is the number of samples and C is an arbitrary positive number added when $X \le 0$, to make log-transformations possible.

In the PLS analyses, the goodness-of-fit parameter Q^2 was used to quantify the model performance, which is the average (n = 7, default value in SIMCA) explained

variance of a randomly selected fraction (1/n of the data) of data not used to fit the model. R^2 and Q^2 are often similar, but the latter will decline as models become increasingly over-fit. PLS models provide coefficients and weights that describe the direction and relative strength of the relationship between X and Y variables; weights with larger absolute values indicate greater importance to a given latent component. All models were refined by iteratively removing variables that had non-significant coefficients. This procedure served to minimize the difference between R^2 and Q^2 values. The relative importance of each X variable was ranked using 'variable importance on the projection' (VIP) scores, derived as the sum of square of the PLS weights across all components. VIP values greater than one are thought to indicate variables that are most important to the overall model (Eriksson et al. 2006).

References

Cohen, J. 1988. *Statistical Power Analysis for the Behavioral Sciences*. Hillsdale: Lawrance Erlbaum Associates.

Costello, A.B., and J.W. Osborne. 2005. Best practices in exploratory factor analysis: Four recommendations for getting the most from your analysis. *Practical Assessment, Research & Evaluation* 10: 1-9. doi: http://pareonline.net/pdf/v10n7.pdf

Eriksson, L., E. Johansson, N. Kettaneh-Wold, J. Trygg, C. Wikström and S. Wold. 2006. Multi- and megavariate data analysis – Part 1: Basic Principles and Applications, Umetrics AB, Umeå.

Faul, F., E. Erdfelder, A. Buchner and A.-G. Lang. 2009. Statistical power analyses using G*Power 3.1: Tests for correlation and regression analyses. *Behavior Research Methods* 41: 1149-1160. doi: 10.3758/brm.41.4.1149

Gauch, H.G., Jr. 1982. *Multivariate Analysis in Community Ecology*. Cambridge: Cambridge University Press.

Geladi, P. and B.R. Kowalski. 1986. Partial least-squares regression - a tutorial. *Analytica Chimica Acta* 185: 1-17. doi: 10.1016/0003-2670(86)80028-9

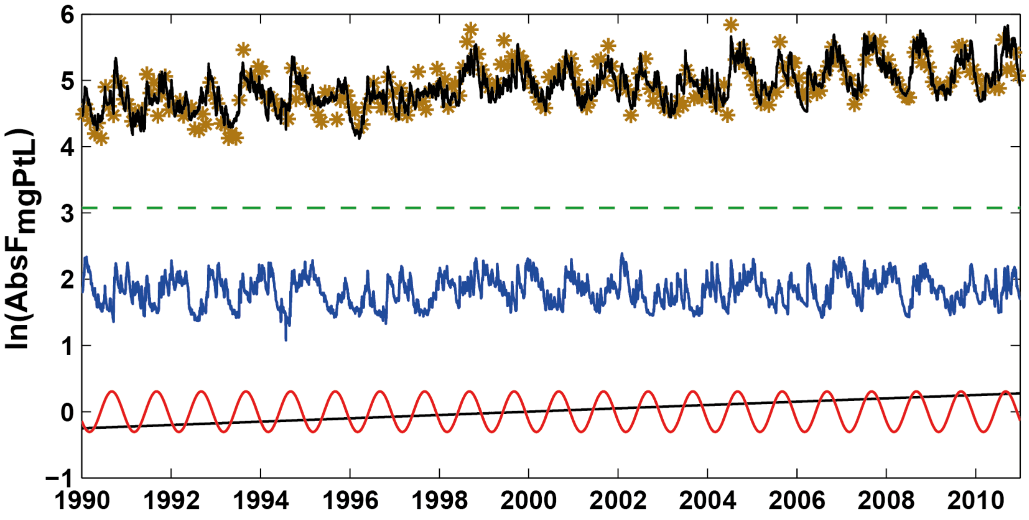

Fig. S1. 21 year time series of empirical and modeled $AbsF_{mgPtL}$ concentration and model terms in one of the watercourses. The intercept (green dashed line), discharge term (blue line), seasonality term (red line) and trend term (black straight line) add up the modelled concentration (black line). The empirical concentration is the brown stars.

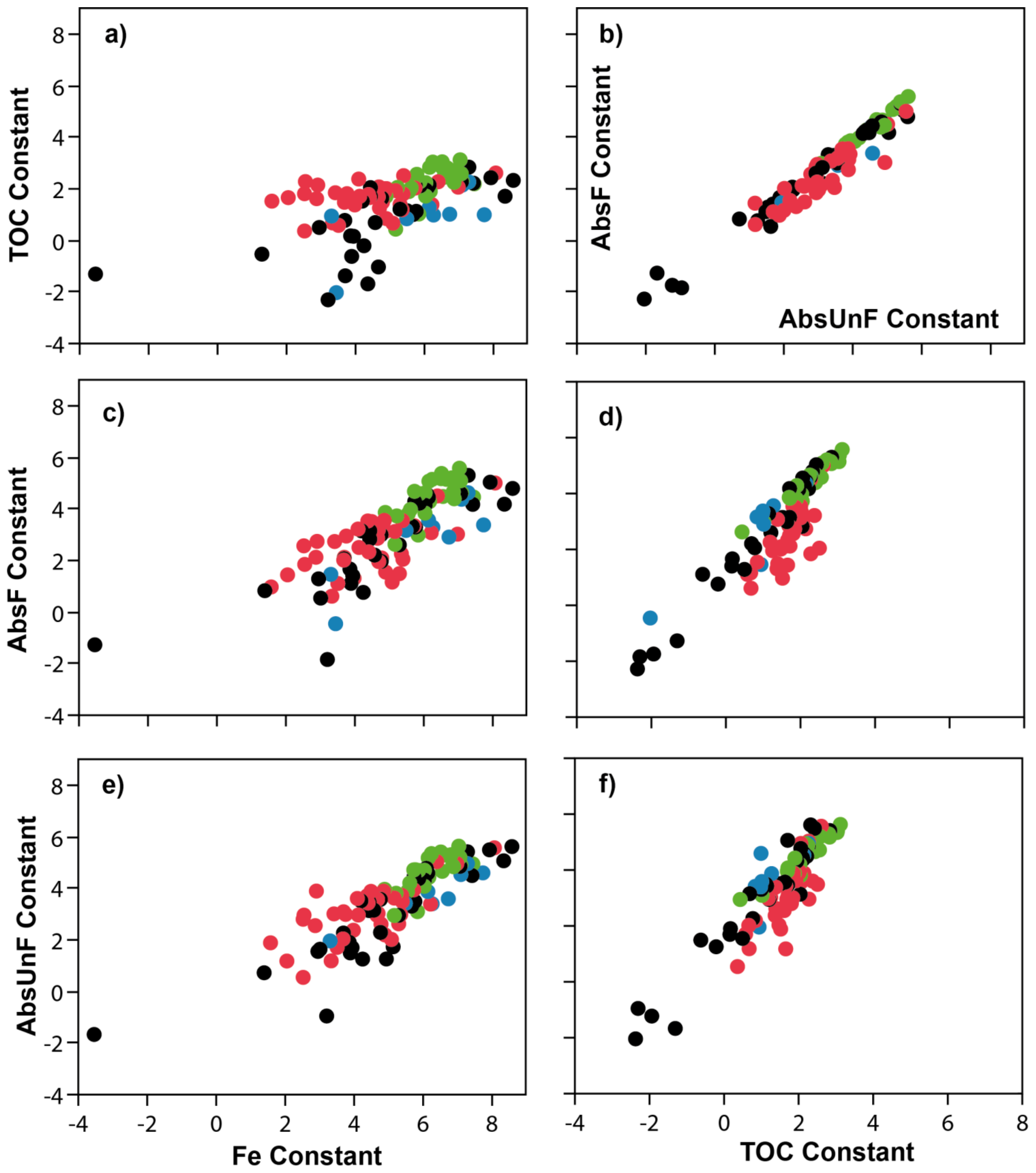

Fig. S2. Correlations between Fluxmaster calculated significant ($p<0.05$) Constant for $AbsF_{mgPtL}$, $AbsUnF_{mgPtL}$, Fe and TOC. The colors on the dots in the graph represent land use (red for agriculture, green for forest, blue for wetland and black for mixed land use).

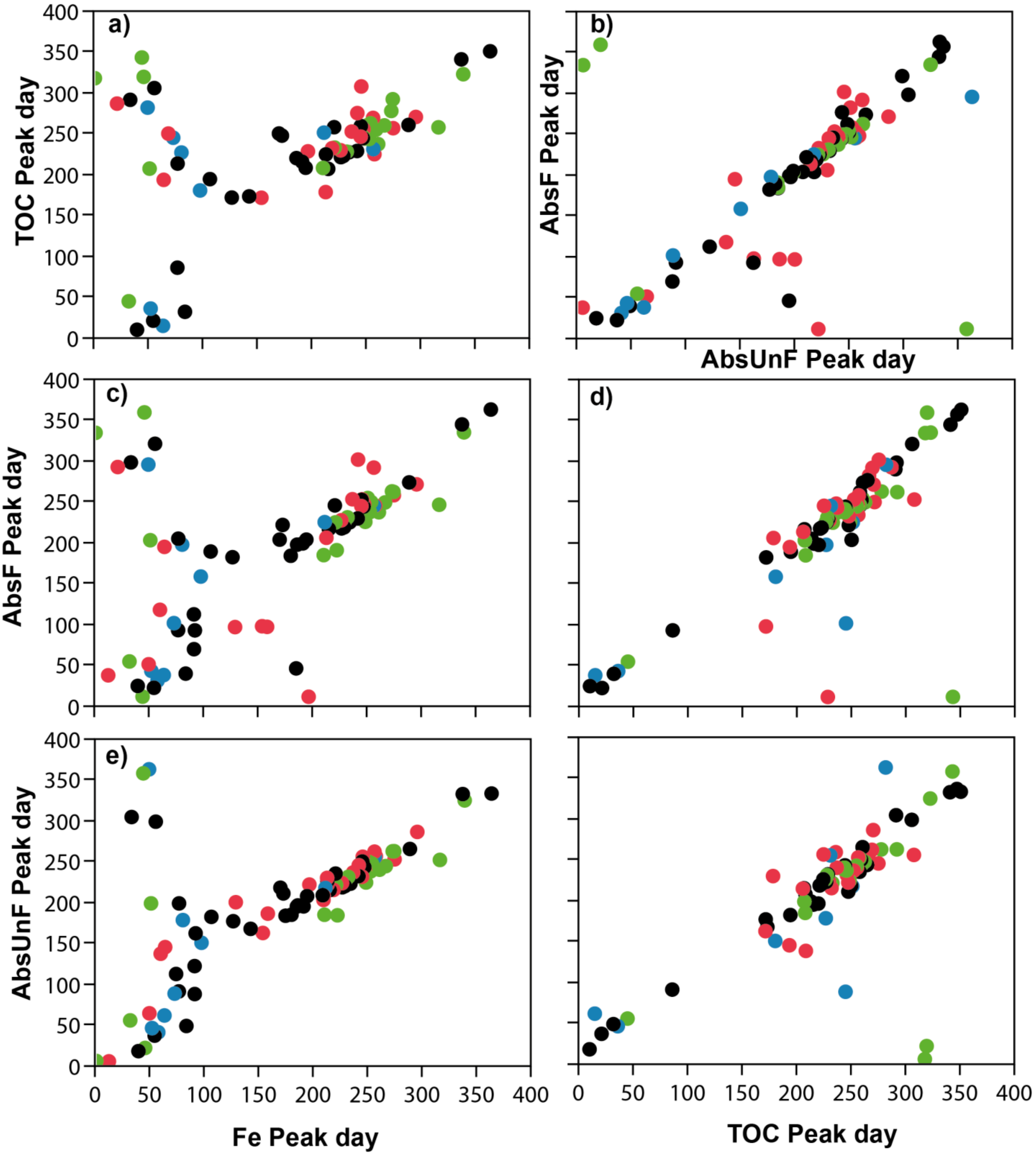

Fig. S3. Correlations between Fluxmaster calculated significant ($p<0.05$) Peak day for $AbsF_{mgPtL}$, $AbsUnF_{mgPtL}$, Fe and TOC. The colors on the dots in the graph represent land use (red for agriculture, green for forest, blue for wetland and black for mixed land use).
